# Lymphocytic Choriomeningitis with Severe Manifestations, Missouri, USA

**DOI:** 10.3201/eid1710.110911

**Published:** 2011-10

**Authors:** Scott Folk, Shari Steinbecker, Joyce Windmeyer, Adam MacNeil, Shelley Campbell, Pierre E. Rollin

**Affiliations:** Heartland Regional Medical Center, St. Joseph, Missouri, USA (S. Folk, S. Steinbecker, J. Windmeyer);; Centers for Disease Control and Prevention, Atlanta, Georgia, USA (A. MacNeil, S. Campbell, P.E. Rollin)

**Keywords:** lymphocytic choriomeningitis, viruses, symptoms, diagnosis, Missouri, letter

**To the Editor:** Lymphocytic choriomeningitis virus (LCMV) is an arenavirus maintained zoonotically in house mice (*Mus musculus*) and may also be carried by pet rodents, especially hamsters (*1**–**3*). Infection of healthy humans usually results in nonspecific febrile illness. However, LCMV infection can cause severe symptoms, including aseptic meningitis (*4*).

Early data suggested <8% of central nervous system manifestations of viral etiology were caused by LCMV (*5*). In contrast, in a more recent study of 91 cases of encephalitis among persons with potential rodent contact, LCMV was not detected (*6*). We describe 2 recent unrelated LCMV infections with central nervous system manifestations, which were associated with rodent exposures, as a reminder that LCMV should be considered in cases of aseptic meningitis of unknown etiology.

In July 2008, an 89-year-old man in Missouri, USA, with a history of hypertension received a prescription for metolazone and was given methotrexate (2.5 mg orally, 3×/wk) because of a pharmacy error. Two weeks later, he showed confusion, speech difficulty, and had a fever. When hospitalized 3 weeks after symptom onset, the patient was drowsy but able to answer questions and had a supple neck, plantar responses in extension, and a temperature of 38.8°C. Methotrexate treatment was then stopped.

Routine laboratory test results were within reference ranges, with the exception of a serum aspartate aminotransferase level of 92 U/L (reference range 0–37 U/L) and an alanine aminotransferase level of 78 U/L (reference range 0–65 U/L). Two blood cultures were sterile, a chest radiograph showed cardiomegaly, and cranial computed tomography without contrast showed moderate cerebral atrophy.

The patient was empirically given intravenous ceftriaxone (1 g) and intravenous azithromycin (500 mg, 1×/d for 7 days). Two days after he was hospitalized, cranial magnetic resonance imaging showed mild-to-moderate, chronic, small vessel ischemia and involutional changes. Serologic test results for HIV and West Nile virus were negative. On the fourth hospital day, lumbar puncture yielded clear, colorless cerebrospinal fluid (CSF) containing 1 erythrocyte and 98 leukocytes/high-powered field (1% neutrophils, 95% lymphocytes, 4% monocytes); protein level was 127 mg/dL (reference range 15–45 mg/dL), and glucose level was 40 mg/dL (reference range 43–70 mg/dL). CSF test results for bacteria, cryptococcal antigen, and herpes simplex virus (HSV) and PCR result for *Borrelia burgdorferi* were negative. CSF and serum submitted to the Centers for Disease Control and Prevention showed LCMV-specific immunoglobulin (Ig) M titers (serum 6,400, CSF 1,280); IgG was not detected. Virus was not isolated.

The patient’s son-in-law reported that mice had been trapped in the patient’s home during the previous winter. The patient was treated supportively and discharged from the hospital 30 days after admission. Six weeks after discharge, the patient was well, without residual neurologic or cognitive deficits.

In November 2010, a 34-year-old woman in Missouri was hospitalized with a 1-day history of progressive headache, neck pain, photophobia, nausea, and vomiting. She had a history of asthma and migraine headaches and had twice undergone surgical repair of congenital heart defects in childhood. She was alert and cooperative, her neck was supple, results of a neurologic examination were normal, and her temperature was 38.6°C.

Laboratory studies showed a hemoglobin level of 11.0 g/dL, a hematocrit of 33.1%, a peripheral leukocyte count of 9,600 cells/mm^3^, a platelet count of 308,000/mm^3^, a serum creatinine level of 0.7 mg/dL, and normal liver functions. A chest radiograph showed mild interstitial changes. Cranial computed tomography without contrast showed normal results. Lumbar puncture showed hazy, colorless CSF, no erythrocytes, 544 leukocytes/high-powered field (11% neutrophils, 84% lymphocytes, 5% monocytes), a protein level of 122 mg/dL, and a glucose level of 56 mg/dL. Results of Gram staining; bacterial culture; and tests for enterovirus, HSV-1, HSV-2 (PCR), and West Nile virus (IgM and IgG) were negative.

The patient was empirically given a 7-day course of ceftriaxone (2 g intravenously 1×/d) and vancomycin (1 g intravenously every 12 hours). CSF submitted to the Centers for Disease Control and Prevention was positive for LCMV by PCR and virus isolation. LCMV-specific IgM (titer 1,280) was detected; IgG was not detected. The patient had observed mice in her home 3 months before hospitalization, although she did not recall any direct contact with them. Her condition improved with supportive therapy and she was released.

These patient observations underscore the potential for severe manifestations of LCMV infection. Neurologic manifestations may mimic those of infections with other viruses, including HSV-1, HSV-2, enteroviruses, and arboviruses, necessitating specific diagnostic tests for identification of the cause, particularly in patients who report recent contact with wild or pet rodents.

The ubiquitous nature of the LCMV reservoir and documented infections in numerous localities in the United States and internationally imply widespread geographic risk for LCMV infection (*7*). Serosurveys in the southern and eastern United States have indicated previous infection in ≈3%–5% of persons (*1**,**8*). However, recent data from upstate New York indicated a seroprevalence <1% (*9*). Thus, the risk for LCMV infection can be minimized by active exclusion and trapping of rodents in the home and avoidance of pet rodents.
